# Investigation of Initial Viral Loads and Patient Characteristics as Predictors of COVID-19 Outcomes: A Retrospective Cohort Study

**DOI:** 10.3390/idr15050057

**Published:** 2023-10-08

**Authors:** Elfira Yusri, Syandrez Prima Putra, Liganda Endo Mahata, Andani Eka Putra

**Affiliations:** 1 Department of Clinical Pathology, Faculty of Medicine, Universitas Andalas, Padang 25163, Indonesia; elfirayusri@med.unand.ac.id; 2 Universitas Andalas Hospital (RS UNAND), Universitas Andalas, Padang 25163, Indonesia; 3 Department of Microbiology, Faculty of Medicine, Universitas Andalas, Padang 25163, Indonesia; andani_ep@yahoo.com; 4 Center for Diagnostic and Research on Infectious Diseases (PDRPI), Faculty of Medicine, Universitas Andalas, Padang 25163, Indonesia; 5 Department of Pharmacology and Therapy, Faculty of Medicine, Universitas Andalas, Padang 25163, Indonesia; ligandaendomahata@med.unand.ac.id

**Keywords:** COVID-19, initial viral load, RT-qPCR, Ct value, outcome

## Abstract

Limited evidence exists on whether initial viral load and patient characteristics can predict unfavorable outcomes in future outbreaks of coronavirus disease 2019 (COVID-19). This retrospective cohort study examined the relationship between the initial viral load, patient characteristics, and outcomes during the second-wave COVID-19 outbreak in West Sumatra, Indonesia. We analyzed the COVID-19 patients admitted to a secondary hospital between the 1 June 2021 and the 31 August 2021. The initial viral load was determined using the real-time quantitative-polymerase chain reaction (RT-qPCR) cycle threshold (Ct) value, categorized as low (LIVL, Ct > 20) or high (HIVL, Ct ≤ 20). Multivariate logistic regression was used to assess the relationship between the initial viral load, age, sex, vaccination status, comorbidities, and outcomes, including disease severity, hospital stay length, ICU admission, invasive ventilation, and in-hospital mortality. Receiver operating characteristic (ROC) curves and the area under the curve (AUC) were used to assess the diagnostic performance of the initial Ct values in predicting COVID-19 outcomes. The study included 373 patients (median age [range]: 48 [0–94]; male: 40.21%; HIVL: 34.85%; unvaccinated: 86.06%; comorbidities: 52.01%). The HIVL patients significantly had a lower risk of developing severe/critical outcomes (OR: 0.506; 95% CI: 0.310–0.825; *p* = 0.006) and needing invasive ventilation (OR: 0.290; CI: 0.098–0.854; *p* = 0.025). The Ct value used to indicate severe/critical outcomes was 23.57. More severe outcomes were significantly observed in LIVL patients, those aged >60 years, males, unvaccinated individuals, and those with comorbidities. This study emphasizes the importance of primary prevention, early screening, and immediate care for COVID-19 in saving lives.

## 1. Introduction

The waves of severe acute respiratory syndrome coronavirus 2 (SARS-CoV-2) infections have significantly impacted global healthcare capacity, leading to increased morbidity and mortality worldwide, particularly before the availability of vaccines [1]. The second-wave of COVID-19 occurred in West Sumatra from June to August 2021, with catastrophic repercussions, including high occurrences and fatality rates. The B.1.617.2 (Delta) variant predominated during this time period [2]. This variant carries several mutations, including T19R, L452R, T478K, D614G, P681R, and d960N in the spike protein, allowing it to spread rapidly compared to the initial Alpha variant [3]. In Indonesia, where only a small percentage of the population was fully vaccinated at the time, the Delta variant caused a significant number of deaths [4]. Although the development of vaccines and the availability of antiviral drugs and neutralizing antibodies have helped mitigate the crisis, the adaptive mutations of the virus continue to pose challenges [5,6]. Therefore, understanding the prognostic value of the initial viral load and the clinical characteristics at diagnosis is crucial for improving therapeutic outcomes, although evidence in this area remains limited.

Reverse-transcription quantitative polymerase chain reaction (RT-qPCR) is the most accurate diagnostic method for COVID-19, assessing the viral load by measuring the cycle threshold (Ct) values of target genes [7]. Despite the known link between lower Ct values (indicating a higher viral load) and poorer clinical outcomes, there are gaps in the understanding due to the variability in the Ct value’s detection timing and differences in viral clearance rates [8,9,10,11]. Longitudinal studies are needed to establish the prognostic value of initial Ct values [12]. Additionally, while patient characteristics such as age, sex, vaccination status, and comorbidities like diabetes and hypertension are associated with higher mortality rates [13,14,15], their interaction with initial Ct values remains understudied, particularly in outbreak scenarios. This study aims to address these gaps by examining the relationship between the initial Ct values, patient characteristic, and treatment outcomes during the second-wave emergence of COVID-19, providing valuable insights for managing future variants.

## 2. Materials and Methods

### 2.1. Study Settings and Population

We conducted a retrospective cohort study at the UNAND Hospital in Padang, Indonesia, during the emergence of the second-wave COVID-19 outbreak. The study included all the patients with RT-qPCR-confirmed COVID-19 who were admitted to the hospital between the 1 June 2021 and the 31 August 2021. The power calculation for the sample size was set at 95%, with a minimal sample of 224. The initial viral loads of the patients were obtained from the PDRPI laboratory, which serves as the central laboratory for COVID-19 diagnosis in the West Sumatra Province. Clinical characteristics such as age, sex, vaccination status, and comorbidities were extracted from the medical records of the UNAND Hospital on the first day of hospital care. The patients with insufficient records were excluded. The outcomes were obtained and analyzed from the hospital information system after a longitudinal observation during hospital treatment.

### 2.2. Data Collection

The initial viral loads were determined based on the Ct value of the SARS-CoV-2 RT-qPCR test from the nasopharyngeal swab taken at the time of the first laboratory-confirmed diagnosis. We selected the lowest Ct value among the targeted SARS-CoV-2 genes (RdRp, N gene, or E gene) and categorized the Ct values as either high (Ct ≤ 20) or low (Ct > 20), as previously described in earlier studies [16,17]. Age was divided into two groups: ≤60 years and >60 years. Sex was categorized as male or female. Vaccination and comorbid status (yes or none) were recorded, regardless of the number of vaccine doses or comorbidities. The outcome variables included the following: (1) disease severity (asymptomatic, mild, moderate, severe, and critical illness based on the NIH criteria [18]); (2) length of hospital stay (≥14 days); (3) ICU admission (yes or none); (4) need for invasive ventilation (yes or none); and (5) in-hospital mortality (yes or none). Data were collected from the 1 January 2022 to the 30 June 2022, and the range of COVID-19 patient data obtained for this study was from the 1 June 2021 to the 31 August 2021. The authors had access to information that could identify individual participants during or after data collection.

### 2.3. Statistical Analysis

We performed statistical analysis using SPSS version 25.0 (SPSS Inc., Chicago, IL, USA). Continuous variables were presented as the median with data range (minimum to maximum) and compared using the Mann–Whitney or Kruskal–Wallis test. Categorical variables were presented as numbers (percentages) and compared using the chi-square test. Multiple logistic regression analyses were conducted to estimate the relationship between the initial viral loads, clinical characteristics, and outcome variables. Odds ratios (ORs) and 95% confidence intervals (CIs) were calculated. The level of significance for each two-sided statistical test was set to 0.05. The diagnostic performance of the initial Ct values for predicting COVID-19 outcomes was analyzed using receiver operating characteristic (ROC) curves and the area under the curve (AUC).

## 3. Results

### 3.1. Initial Viral Loads and Patient Characteristics

A total of 373 patients were included in this study (Supplementary Table S1). The patient characteristics, stratified according to the high initial viral load (HIVL; Ct ≤ 20) and low initial viral load (LIVL; Ct > 20) groups, are summarized and compared in Table 1. The initial Ct values differed significantly between the two groups (HIVL: median [min–max], 16.66 [9.80–19.80] vs. LIVL: median [min–max], 27.02 [20.03–36.79]; *p* < 0.001). The proportion of unvaccinated patients was significantly higher in the LIVL group compared to the HIVL group (90.95% vs. 76.92%, *p* < 0.001). Furthermore, a significantly higher proportion of patients in the LIVL group had comorbidities compared to the HIVL group (58.44% vs. 40.00%, *p* = 0.001).

### 3.2. Disease Severity

A higher number of cases was reported in the moderate group of COVID-19 patients (106, 28.42%) compared to other severity groups. A significantly higher proportion of severe and critical outcomes was reported among patients aged >60 years (severe: 40.4%, critical: 35.7%; *p* < 0.001), male (severe: 53.9%; critical: 57.1%; *p* < 0.001), unvaccinated (severe: 98.9%; critical: 96.4%; *p* < 0.001), patients with comorbidities (severe: 68.5%; critical: 71.4%; *p* < 0.001), and LIVL patients (severe: 71.9%; critical: 85.7%, respectively; *p =* 0.045) (Table 2).

### 3.3. Length of Hospital Stay

The length of hospital stays (days from first admission to discharge) in COVID-19 patients is summarized to examine its relationship with the clinical characteristics and initial viral load. Most patients had in-hospital treatment for less than 14 days (307, 82.31%), regardless of their discharge status (recovered, referred, or deceased). This result indicates no significant difference between the clinical characteristics, the initial viral load, and the length of hospital stay (Table 3).

### 3.4. ICU Admission

The proportion of ICU admissions was 23.32%. The prevalence of >60 years old patients admitted to the ICU was significantly higher than those who were not (44.83% vs. 24.83%, *p* < 0.001). Similar findings were also observed in males (55.17% vs. 35.66%, *p* = 0.001) and patients with comorbidities (65.52% vs. 47.90%, *p* = 0.004). A significantly higher proportion of unvaccinated patients needed ICU admission (98.85% vs. 82.17%, *p* < 0.001). However, there was no significant relationship between the initial viral load and the probability of entering the ICU (Table 4).

### 3.5. Invasive Ventilation

The proportion of patients requiring invasive ventilation was 7.51%, with a median age of 57 years (range 27–80) (*p* = 0.003). There was a significantly higher percentage of patients with comorbidities in this group compared to the non-ventilated group (71.43% vs. 50.43%, *p* = 0.032). Additionally, a significantly higher proportion of HIVL patients did not require invasive ventilation (36.52% vs. 14.29%, *p* = 0.018) (Table 5).

### 3.6. In-Hospital Mortality

The case fatality rate was 11.26%, and the median age (range) of the deceased patients was 58.5 (35–84) (*p* < 0.001). There was a significantly higher proportion of deceased patients in the >60 years age group (45.2% vs. 27.5%, *p* = 0.018). Furthermore, a higher percentage of male patients were found among the deceased (59.5% vs. 37.8%, *p* = 0.007). All the vaccinated patients (15.7%) survived and none of them died (*p* = 0.006). Additionally, a higher proportion of deceased patients had comorbidities compared to the survivors (81.0% vs. 48.3%, *p* < 0.001). There was no significant relationship between the initial viral load and the in-hospital mortality (Table 6).

### 3.7. Factors Associated with the Outcomes

We presented the multivariate logistic regression model (unadjusted) to address the factors associated with the outcomes (Table 7). The factors associated with severe/critical symptoms were the following: age > 60 years old (odds ratio [OR], 2.323; 95% confidence interval [CI], 1.391–3.878; *p* = 0.001); male (OR, 1.974; 95% CI, 1.212–3.216; *p* = 0.006); unvaccinated (OR, 13.461; 95% CI, 3.117–58.131; *p* < 0.001); and patient with comorbidities (OR, 2.286; 95% CI, 1.381–3.784; *p* = 0.001). No significant relationship existed between all the variables and the length of hospital stay > 14 days. The factors associated with ICU admission were the following: age > 60 years (OR, 1.881; 95% CI, 1.101–3.215; *p* = 0.021); male (OR, 1.891; 95% CI, 1.129–3.168; *p* = 0.016); and unvaccinated patient (OR, 17.680; 95% CI, 2.368–131.995; *p* = 0.005). Lastly, male patients (OR, 2.010; 95% CI, 1.010–3.999; *p* = 0.047) and patients with comorbidities were significantly associated with the in-hospital mortality rates (OR, 3.788; 95% CI 1.656–8.664; *p* = 0.002).

### 3.8. Relationship between the Initial Viral Load and the Outcomes

**Figure 1:**
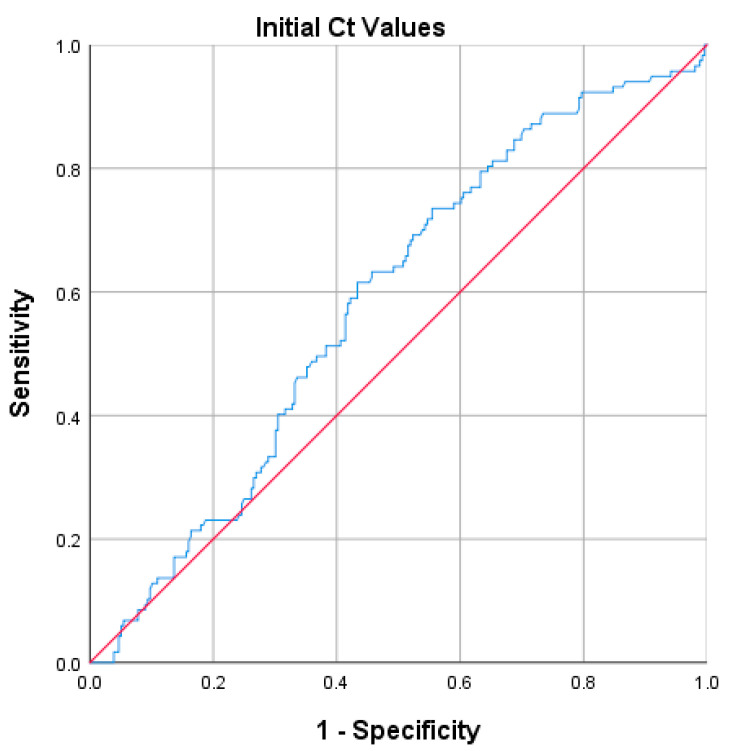
The receiver operating characteristic (ROC) curve shows the diagnostic performance of initial viral load (Ct value) in predicting severe/critical outcomes of COVID-19 patients. The area under the curve (AUC) is 0.583 (95% confidence interval [CI], 0.523–0.643, *p* = 0.010).

To determine the relationship between the initial viral load and the COVID-19 outcomes, we performed an adjusted regression model of initial viral load for each outcome category (Table 8). This analysis showed that the HIVL patients significantly had lower odds of developing severe/critical symptoms (OR, 0.506; 95% CI, 0.310–0.825, *p* = 0.006) and lower odds of requiring invasive ventilation (OR, 0.290; 95% CI, 0.098–0.854; *p* = 0.025). Generally, the proportions of the HIVL group suggested better outcomes than the LIVL group. The diagnostic performance of the initial Ct value in predicting severe/critical symptoms was significantly observed at Ct 23.57 (sensitivity, 61.5%; specificity, 56.6%; *p* = 0.010) (Figure 1).

## 4. Discussion

The viral load among COVID-19 patients with different characteristics has been investigated in several studies to predict treatment outcomes [19,20,21,22]. However, there is inadequate information on the relationship between the initial viral load—the first-diagnosed RT-qPCR Ct value obtained from the nasopharyngeal swab—, the patient characteristics, and the outcomes of SARS-CoV-2 infection. Our study evaluates the capability of the initial viral load to explore COVID-19 prognosis in conjunction with the characteristic of the patients during the Delta variant’s emergence. After RT-qPCR-confirmed diagnosis, the availability of initial Ct values emphasizes possibilities to determine the patient’s prognosis following hospital treatment. Here, we observe a significant relationship between a low initial viral load (LIVL, Ct > 20), older age, male sex, unvaccinated status, and patients with comorbidities and the more severe outcomes.

In our study, most COVID-19 patients were first admitted with a significantly high viral load with Ct ≤ 20 (HIVL). Of those, a considerably higher proportion was vaccinated and without comorbidities, compared to the LIVL group. We assume that early disease detection explains this finding, where the viral load around the onset is high [23]. Nevertheless, following the government policy to expand mass screening, there are possibilities that most vaccinated and previously healthy patients without a disease history will seek or accept medical treatment immediately at the early onset of the disease. However, this finding shows that the transmission of the SARS-CoV-2 dominated by the Delta variant in the vaccinated population remains a concern, as reported in a previous study [24].

In our cohort, the severe and critical outcome of COVID-19 patients was significantly associated with older age, male sex, unvaccinated status, and comorbidities, as reported in several studies [25,26]. Older patients risk severe and critical symptoms due to ageing-related chronic pro-inflammatory status that leads to the alteration of the immune system against the pathogen. A similar reason is also explained in patients with pre-existing comorbidities, in line with a faster COVID-19 progression due to prior organ damage. In males, the systemic spread of SARS-CoV-2 may be facilitated by normal serum testosterone levels that encourage viral entry into the host cells, as well as the higher ACE2 levels on the endothelium of the pulmonary vessels. Additional infection-predisposition lifestyles (such as smoking) should also be considered in male patients [27]. We also observed a significant association between the initial viral load and the severity, where the LIVL group (Ct > 20) suggested poorer symptoms than the HIVL (Ct ≤ 20) group. This finding should answer the unclear relation observed in a previous study, explaining that acute respiratory distress syndrome (ARDS), the severe to critical stage of COVID-19, occurs at the disease’s later stage, when the viral load is low [28].

The length of hospital stays (LOS) for COVID-19 in our study was in line with most studies outside China, with a median shorter than 14 days [29]. Our investigation did not find a correlation between the initial viral load, the patient characteristics, and the LOS. In a previous study, the viral load on admission was higher in patients with LOS ≥ 10 days than in patients with LOS < 10 days. Only age was associated with an increased LOS in COVID-19 patients [30]. In contrast, another study in the United States reported that a higher initial viral load was associated with a shorter duration of the symptoms [12]. The possible hypothesis for this difference should include another essential factor, especially the appearance of pneumonia confirmed in the initial computed tomography [31]. This factor should be considered when projecting cost-effective treatment of the patients in a hospital.

Near a quarter of patients in our study needed ICU support, especially for the >60 years old, male, unvaccinated, and comorbid-positive groups. A recent study also reported a similar finding in the Delta variant at the peak of the cases, confirming the critical role of individual characteristics, vaccination status, and comorbidities in determining COVID-19 outcomes [32]. The same pattern is also observed in COVID-19 mortality, similar to the finding in Tehran during the Delta surge [33]. In our analysis, we have taken into consideration the unique age and sex profiles associated with the Delta variant, as suggested by previous research [34]. Notably, our findings align with previous observations, indicating that there is a peak in COVID-19 mortality around the age group of 50–59, consistent with the patterns observed for Delta and other variants.

Furthermore, no association was observed between the initial viral load, ICU admission, and in-hospital mortality. Instead, this cohort revealed the critical connection between the initial viral load and the need for invasive ventilation, where the LIVL group tended to have a higher risk than the HIVL group. In our recent study, we identified a significant association between the initial viral load and the requirement for invasive ventilation, differently from previous studies [35,36]. In line with the same finding in patients within the severe and critical groups, we assumed that, if the patients had been admitted to the hospital with a low viral load at the beginning, they were already at the later stage [30] of the disease and, thus, tended to have more severe symptoms that would lead to ARDS, which then required invasive ventilation in the nearly fatal cases [37]. In contrast, there was a probability of the HIVL group receiving immediate treatment that prevented them from having critical outcomes.

A multivariate logistic regression model was conducted in this retrospective cohort study to address the factors associated with COVID-19 outcomes. In general, poorer outcomes were significantly observed in patients with older age, male sex, no vaccination, underlying comorbidities, and LIVL at early diagnosis. We hypothesize that the result of RT-qPCR examination at a particular time, as the indicator of the viral load, may, in this case, express the disease’s stage. In addition to a lower viral load [38], a recent study explains that, at the late phase of the disease, the pro-inflammatory cytokine IL6 and tumor necrosis factor (TNF)-α levels are proportional, contrasting with the number of T cells. The use of drugs inhibiting virus replication, cytokine levels, and immunosuppressant drugs at the early stage of the disease (HIVL) should prevent severe/critical outcomes in most COVID-19 patients, thus decreasing the mortality rate [39]. Furthermore, according to our findings, the initial Ct value threshold for predicting a severe/critical outcome was 23.57. Given the association between the initial viral loads and the outcomes, the Ct value offers a predictive relevance for the development of severe/critical COVID-19. The sensitivity and specificity, however, were modest.

Our study has limitations due to the use of retrospective data, which may have introduced biases in the collection process. Additionally, we did not differentiate the primers used in SARS-CoV-2 RT-qPCR detection, and it is possible that they may affect the sensitivity or specificity for detecting the Delta variant. Moreover, the limited number of samples in terms of vaccination coverage and doses at the collection dates may have introduced biases in the data analyses, as we only included a single parameter for vaccination (yes or none). However, despite these limitations, our study highlights the initial factors that can be predictive of the final outcomes of COVID-19 patients in outbreak settings.

## 5. Conclusions

This retrospective cohort study elucidates the potential impact of initial viral load and patient characteristics on determining COVID-19 outcomes and prognosis, particularly in the context of outbreaks. A patient with a high viral load at the early hospital treatment stage (Ct ≤ 20) had better outcomes than a patient with a low one, potentially due to immediate detection and patient care near the onset of the disease. Older age, male sex, unvaccinated, and comorbid-positive patients were significantly associated with poorer outcomes. The utilization of the initial Ct value obtained from the nasopharyngeal swab RT-qPCR as a predictive tool in COVID-19 prognosis must be evaluated due to its poor sensitivity and specificity. The findings from this research may not only enhance our understanding of current disease dynamics but also contribute valuable insights to inform future pandemic preparedness and management strategies.

## Figures and Tables

**Table 1:** Relationship between patient characteristics and initial viral loads.

Characteristic	Frequency	HIVL (Ct ≤ 20)	LIVL (Ct > 20)	*p*-Value
Age (years)	48 (0–94)	38.5 (0–94)	50 (0–91)	0.114
Age > 60 years	110 (29.49%)	37 (28.46%)	73 (30.04%)	0.750
Male	150 (40.21%)	48 (36.92%)	102 (41.98%)	0.343
Unvaccinated	321 (86.06%)	100 (76.92%)	221 (90.95%)	<0.001 *
With comorbidities	194 (52.01%)	52 (40.00%)	142 (58.44%)	0.001 *
Initial Ct value	23.40 (9.80–36.79)	16.66 (9.80–19.80)	27.02 (20.03–36.79)	<0.001 *
Total	373	130	243	

* Statistically significant; HIVL, high initial viral load; and LIVL, low initial viral load.

**Table 2:** Relationship between patient characteristics, initial viral load, and severity.

Characteristic	Severity					*p*-Value
	Asymptomatic	Mild	Moderate	Severe	Critical	
Age (years)	37 (8–74)	27 (0–72)	52 (0–79)	60 (18–94)	57 (27–80)	<0.001 *
Age > 60 years	14 (23.7%)	7 (7.7%)	35 (33.0%)	44 (49.4%)	10 (35.7%)	<0.001 *
Male	22 (37.3%)	18 (19.8%)	46 (43.4%)	48 (53.9%)	16 (57.1%)	<0.001 *
Unvaccinated	54 (91.5%)	71 (78.0%)	81 (76.4%)	88 (98.9%)	27 (96.4%)	<0.001 *
With comorbidities	6 (10.2%)	35 (38.5%)	72 (67.9%)	61 (68.5%)	20 (71.4%)	<0.001 *
Initial Ct value	23.10 (11.62–34.19)	21.86 (9.80–36.79)	22.44 (11.23–36.26)	24.44 (10.75–33.63)	25.31 (10.93–33.12)	0.105
HIVL (Ct ≤ 20)	22 (37.3%)	38 (41.8%)	41 (38.7%)	25 (28.1%)	4 (14.3%)	0.045 *
Total	59	91	106	89	28	

* Statistically significant; and HIVL, high initial viral load.

**Table 3:** Relationship between patient characteristics, initial viral load, and length of hospital stay.

Characteristic	Length of Hospital Stay		*p*-Value
	≤14 Days	>14 Days	
Age (years)	47 (0–94)	52.5 (3–91)	0.163
>60 years	221 (71.99%)	42 (63.64%)	0.177
Male	118 (38.44%)	32 (48.48%)	0.131
Unvaccinated	262 (85.34%)	59 (89.39%)	0.389
With comorbidities	158 (51.47%)	36 (54.55%)	0.650
Initial Ct value	23.10 (9.80–36.79)	23.87 (11.23–36.26)	0.536
HIVL (Ct ≤ 20)	196 (63.84%)	47 (71.21%)	0.254
Total	307	66	

**Table 4:** Relationship between patient characteristics, initial viral load, and ICU admission.

Characteristic	ICU Admission		*p*-Value
	None	Yes	
Age (years)	41 (0–87)	59 (18–94)	<0.001 *
Age > 60 years	71 (24.83%)	39 (44.83%)	<0.001 *
Male	102 (35.66%)	48 (55.17%)	0.001 *
Unvaccinated	235 (82.17%)	86 (98.85%)	<0.001 *
With comorbidities	137 (47.90%)	57 (65.52%)	0.004 *
Initial Ct value	23.10 (9.80–36.79)	24.33 (10.75–33.63)	0.158
HIVL (Ct ≤ 20)	106 (37.06%)	24 (27.59%)	0.104
Total	286	87	

* Statistically significant; and HIVL, high initial viral load.

**Table 5:** Relationship between patient characteristics, initial viral load, and invasive ventilation.

Characteristic	Invasive Ventilation		*p*-Value
	None	Yes	
Age (years)	47 (0–94)	57 (27–80)	0.003
Age > 60 years	100 (28.99%)	10 (35.71%)	0.453
Male	134 (38.84%)	16 (57.14%)	0.057
Unvaccinated	294 (85.22%)	27 (96.43%)	0.152
With comorbidities	174 (50.43%)	20 (71.43%)	0.032 *
Initial Ct value	23.10 (9.80–36.79)	25.31 (10.93–33.12)	0.053
HIVL (Ct ≤ 20)	126 (36.52%)	4 (14.29%)	0.018 *
Total	345	28	

* Statistically significant; and HIVL, high initial viral load.

**Table 6:** Relationship between patient characteristics, initial viral load, and in-hospital mortality.

Characteristic	In-Hospital Mortality		*p*-Value
	Discharged/Referred	Deceased	
Age (years)	45 (0–94)	58.5 (35–84)	<0.001 *
Age > 60 years	91 (27.5%)	19 (45.2%)	0.018 *
Male	125 (37.8%)	25 (59.5%)	0.007 *
Unvaccinated	279 (84.3%)	42 (100.0%)	0.006 *
With comorbidities	160 (48.3%)	34 (81.0%)	<0.001 *
Initial Ct value	23.12 (9.80–36.79)	24.09 (12.14–33.12)	0.162
HIVL (Ct ≤ 20)	121 (36.6%)	9 (21.4%)	0.053
Total	331	42	

* Statistically significant; and HIVL, high initial viral load.

**Table 7:** Multivariate logistic regression models of factors associated with COVID-19 outcomes.

Characteristic	Severe/Critical Symptom		Length of Hospital Stay > 14 Days		ICU Admission		Invasive Ventilation		In-Hospital Mortality	
	OR (95% CI)	*p*-Value	OR (95% CI)	*p*-Value	OR (95% CI)	*p*-Value	OR (95% CI)	*p*-Value	OR (95% CI)	*p*-Value
Age > 60 years	2.323 (1.391–3.878)	0.001 *	1.351 (0.749–2.436)	0.317	1.881 (1.101–3.215)	0.021 *	1.018 (0.437–2.371)	0.966	1.472 (0.735–2.949)	0.275
Male	1.974 (1.212–3.216)	0.006 *	1.399 (0.806–2.428)	0.233	1.891 (1.129–3.168)	0.016 *	1.826 (0.816–4.086)	0.143	2.010 (1.010–3.999)	0.047 *
Unvaccinated	13.461 (3.117–58.131)	<0.001 *	1.284 (0.541–3.049)	0.571	17.680 (2.368–131.995)	0.005 *	3.424 (0.443–26.459)	0.238	N/A	N/A
With comorbidities	2.286 (1.381–3.784)	0.001 *	0.965 (0.551–1.690)	0.901	1.690 (0.989–2.890)	0.055	1.962 (0.816–4.716)	0.132	3.788 (1.656–8.664)	0.002 *
HIVL (Ct ≤ 20)	0.703 (0.408–1.212)	0.205	0.748 (0.409–1.368)	0.346	0.895 (0.506–1.586)	0.705	0.384 (0.127–1.163)	0.091	0.751 (0.332–1.701)	0.493

* Statistically significant; OR, odds ratio; CI, confidence interval; HIVL, high initial viral load; and N/A, data not available.

**Table 8:** Adjusted regression model of initial viral load and COVID-19 outcomes.

Viral Load (Adjusted)	N (%) ^1^	OR (95% CI)	*p*-Value
Severe/critical symptoms			
LIVL (Ct > 20)	88 (36.2%)	1	
HIVL (Ct ≤ 20)	29 (22.3%)	0.506 (0.310–0.825)	0.006 *
Hospital stay >14 days			
LIVL (Ct > 20)	47 (19.3%)	1	
HIVL (Ct ≤ 20)	19 (14.6%)	0.714 (0.399–1.277)	0.256
ICU admission			
LIVL (Ct > 20)	63 (25.9%)	1	
HIVL (Ct ≤ 20)	24 (18.5%)	0.647 (0.382–1.097)	0.106
Invasive ventilation			
LIVL (Ct > 20)	24 (9.9%)	1	
HIVL (Ct ≤ 20)	4 (3.1%)	0.290 (0.098–0.854)	0.025 *
In-hospital mortality			
LIVL (Ct > 20)	33 (13.6%)	1	
HIVL (Ct ≤ 20)	9 (6.9%)	0.473 (0.219–1.023)	0.057

^1^ percentage within viral load categories; * statistically significant; OR, odds ratio; CI, confidence interval; HIVL, high initial viral load; and LIVL, low initial viral load.

## Data Availability

All relevant data are within the manuscript and its supporting information files.

## Supplementary Materials

The following supporting information can be downloaded at: https://www.mdpi.com/article/10.3390/idr15050057/s1, Table S1: Patients and variables included in the study.
