# Supplementary material for: Investigation of Initial Viral Loads and Patient Characteristics as Predictors of COVID-19 Outcomes: A Retrospective Cohort Study
Source: Infect Dis Rep. 2023 Oct 8;15(5):589–99. doi: 10.3390/idr15050057 (PMC10606841; doi:10.3390/idr15050057)
Supplement: Supplementary file 1 [file idr-15-00057-s001.zip › idr-2515159-supplementary.pdf]

Supplementary Table S1: Patients and variables included in the study

PATIENT NUMBER	AGE (YEARS)	AGE GROUP (1) > 60 (2) <=60	SEX (1) MALE (2) FEMALE	CT VALUES AT INITIAL DIAGNOSIS	CT VALUES CATEGORY (1) HIGH VIRAL LOAD (<=20) (2) LOW VIRAL LOAD (>20)	VACCINATION (1) NONE (2) YES	COMORBID (1) YES (2) NONE	CLINICAL SYMPTOM (0) ASYMPTOMATIC (1) MILD (2) MODERATE (3) SEVERE (4) CRITICAL	SEVERITY (0) NON SEVERE/CRITICAL (1) SEVERE/CRITICAL	LENGTH OF TREATMENT (0) ≤ 14 DAYS (1) > 14 DAYS	ICU ADMISSION (0) NO (1) YES	INVASIVE VENTILATION (0) NO (1) YES	MORTALITY (0) SURVIVE (1) DEAD
1	22	2	2	14.19	1	2	2	1	0	1	0	0	0
2	68	1	2	32.19	2	1	2	3	1	0	1	0	1
3	26	2	2	30.14	2	1	2	3	1	0	1	0	0
4	0	2	2	34.93	2	2	1	1	0	0	0	0	0
5	66	1	2	21.84	2	1	1	3	1	0	1	0	1
6	27	2	2	12.74	1	1	2	1	0	0	0	0	0
7	60	2	1	15.54	1	1	2	0	0	0	0	0	0
8	56	2	2	25.61	2	1	1	3	1	0	1	0	0
9	29	2	1	23.10	2	1	1	1	0	0	0	0	0
10	62	1	1	29.79	2	1	1	3	1	0	1	0	0
11	58	2	1	21.18	2	1	1	2	0	1	0	0	0
12	52	2	2	32.20	2	1	1	2	0	1	0	0	0
13	18	2	1	26.99	2	1	1	3	1	1	1	0	0
14	23	2	1	27.52	2	1	2	0	0	1	0	0	0
15	23	2	2	22.42	2	1	2	1	0	0	0	0	0
16	35	2	2	16.40	1	2	1	1	0	0	0	0	0
17	27	2	2	32.46	2	1	1	1	0	0	0	0	0
18	73	1	1	33.76	2	1	1	2	0	0	0	0	0
19	30	2	1	11.62	1	1	1	0	0	0	0	0	0
20	64	1	2	28.39	2	1	1	3	1	1	1	0	0
21	20	2	2	32.23	2	2	2	1	0	0	0	0	0
22	73	1	1	21.79	2	1	1	3	1	0	1	0	1
23	62	1	1	26.44	2	1	1	3	1	1	1	0	0
24	59	2	1	25.90	2	1	2	0	0	0	0	0	0
25	54	2	1	32.58	2	1	1	3	1	0	1	0	0
26	70	1	2	15.10	1	1	1	2	0	0	0	0	0
27	21	2	2	15.53	1	2	2	1	0	0	0	0	0
28	60	2	2	21.40	2	1	1	4	1	0	1	1	1
29	52	2	2	27.82	2	2	1	1	0	0	0	0	0
30	24	2	1	32.42	2	1	2	2	0	0	0	0	0
31	24	2	1	19.37	1	2	1	2	0	0	0	0	0
32	53	2	1	26.10	2	1	1	4	1	0	1	1	1
33	63	1	1	21.36	2	1	1	3	1	0	1	0	1
34	83	1	1	21.10	2	1	1	3	1	0	1	0	0
35	25	2	2	32.51	2	1	1	1	0	0	0	0	0
36	24	2	1	25.39	2	1	2	0	0	1	0	0	0
37	26	2	2	31.24	2	1	1	1	0	0	0	0	0
38	29	2	2	17.14	1	1	2	1	0	0	0	0	0
39	26	2	2	30.06	2	1	1	1	0	0	0	0	0
40	63	1	2	17.96	1	1	1	2	0	0	0	0	0
41	21	2	2	13.87	1	1	2	2	0	1	0	0	0
42	27	2	1	32.11	2	1	1	2	0	0	0	0	0
43	22	2	2	22.98	2	1	2	1	0	1	0	0	0
44	27	2	1	20.80	2	1	2	1	0	1	0	0	0
45	52	2	2	26.37	2	1	1	2	0	0	0	0	0
46	32	2	2	22.43	2	1	1	1	0	0	0	0	0
47	71	1	2	24.65	2	2	1	2	0	0	0	0	0
48	43	2	2	19.06	1	2	1	1	0	1	0	0	0
49	20	2	1	19.48	1	2	2	1	0	0	0	0	0

50	45	2	2	28.49	2	1	1	1	0	0	0	0	0
51	24	2	2	15.08	1	1	1	1	0	0	0	0	0
52	37	2	2	18.63	1	1	2	3	1	0	1	0	0
53	38	2	1	33.63	2	1	1	3	1	0	1	0	0
54	28	2	2	31.09	2	1	1	1	0	0	0	0	0
55	53	2	2	15.46	1	1	2	3	1	1	1	0	0
56	27	2	2	28.21	2	1	1	1	0	0	0	0	0
57	27	2	2	17.34	1	1	2	2	0	1	0	0	0
58	54	2	2	20.03	2	2	1	2	0	0	0	0	0
59	88	1	1	22.03	2	1	1	3	1	0	1	0	0
60	25	2	2	22.50	2	1	2	0	0	0	0	0	0
61	24	2	2	29.15	2	1	2	1	0	0	0	0	0
62	69	1	1	31.32	2	1	1	3	1	0	1	0	1
63	33	2	2	16.03	1	2	2	1	0	0	0	0	0
64	62	1	1	22.53	2	1	1	3	1	1	1	0	0
65	51	2	1	33.57	2	2	1	2	0	0	0	0	0
66	19	2	1	20.31	2	1	1	1	0	0	0	0	0
67	22	2	2	16.55	1	1	2	1	0	0	0	0	0
68	29	2	2	31.5	2	1	1	1	0	0	0	0	0
69	60	2	1	21.4	2	1	1	2	0	1	0	0	0
70	77	1	1	19.80	1	1	2	3	1	0	1	0	1
71	27	2	1	23.50	2	1	1	1	0	0	0	0	0
72	26	2	1	24.90	2	1	2	1	0	0	0	0	0
73	61	1	2	23.40	2	1	1	1	0	1	0	0	0
74	36	2	1	27.58	2	1	2	0	0	0	0	0	0
75	62	1	2	29.15	2	2	1	2	0	0	0	0	0
76	71	1	2	27.61	2	1	1	2	0	1	0	0	0
77	57	2	1	28.96	2	1	2	0	0	0	0	0	0
78	26	2	2	17.53	1	2	2	0	0	0	0	0	0
79	28	2	2	20.67	2	1	2	0	0	0	0	0	0
80	44	2	2	29.75	2	1	2	0	0	1	0	0	0
81	29	2	2	23.64	2	1	1	3	1	0	1	0	0
82	17	2	2	21.17	2	2	2	1	0	0	0	0	0
83	77	1	1	20.60	2	1	2	2	0	0	0	0	0
84	56	2	1	21.51	2	1	2	2	0	0	0	0	0
85	9	2	2	17.07	1	1	2	0	0	0	0	0	0
86	31	2	2	34.19	2	1	2	0	0	0	0	0	0
87	68	1	1	10.75	1	1	2	3	1	0	1	0	0
88	48	2	1	28.3	2	1	1	3	1	1	1	0	0
89	51	2	2	28.49	2	1	1	3	1	0	1	0	0
90	27	2	2	27.39	2	2	2	2	0	0	0	0	0
91	60	2	1	20.93	2	1	1	3	1	0	1	0	0
92	61	1	2	17.11	1	2	1	2	0	1	0	0	0
93	28	2	2	29.02	2	1	2	1	0	0	0	0	0
94	33	2	2	29.02	2	1	2	0	0	0	0	0	0
95	27	2	2	29.06	2	1	2	0	0	0	0	0	0
96	56	2	2	18.70	1	1	2	1	0	0	0	0	0
97	72	1	2	22.46	2	1	2	1	0	0	0	0	0
98	53	2	2	27.15	2	1	2	3	1	0	1	0	1
99	56	2	2	14.15	1	1	1	2	0	0	0	0	0
100	41	2	2	23.68	2	1	1	1	0	0	0	0	0
101	49	2	2	30.35	2	1	1	1	0	0	0	0	0
102	61	1	1	17.54	1	1	1	1	0	0	0	0	0
103	61	1	2	11.61	1	1	2	3	1	0	1	0	0
104	57	2	2	22.25	2	1	2	2	0	0	0	0	0
105	56	2	2	18.28	1	1	1	2	0	0	0	0	0
106	44	2	2	28.83	2	1	1	2	0	0	0	0	0
107	36	2	2	30.40	2	1	1	1	0	1	0	0	0
108	70	1	2	26.45	2	1	1	2	0	0	0	0	0

109	63	1	1	23.64	2	1	1	4	1	1	1	1	1
110	22	2	2	26.09	2	1	2	1	0	0	0	0	0
111	3	2	2	28.55	2	1	2	2	0	1	0	0	0
112	25	2	2	20.13	2	1	2	0	0	0	0	0	0
113	10	2	2	17.02	1	1	2	0	0	0	0	0	0
114	55	2	2	30.21	2	1	1	3	1	0	1	0	1
115	24	2	2	15.17	1	1	2	1	0	0	0	0	0
116	35	2	2	15.19	1	1	2	1	0	0	0	0	0
117	63	1	1	18.15	1	1	1	3	1	0	1	0	1
118	29	2	1	28.05	2	1	1	3	1	1	1	0	0
119	56	2	1	29.87	2	1	2	4	1	0	1	1	0
120	59	2	2	25.12	2	1	2	0	0	1	0	0	0
121	62	1	1	20.69	2	1	1	2	0	0	0	0	0
122	35	2	1	22.02	2	1	1	3	1	0	1	0	1
123	62	1	2	12.71	1	1	1	2	0	0	0	0	0
124	65	1	1	23.10	2	1	2	0	0	0	0	0	0
125	44	2	1	23.10	2	1	1	2	0	0	0	0	0
126	53	2	1	17.02	1	2	1	2	0	0	0	0	0
127	66	1	1	12.54	1	1	2	3	1	0	1	0	0
128	36	2	2	36.26	2	2	2	2	0	1	0	0	0
129	38	2	1	25.43	2	1	1	2	0	1	0	0	0
130	46	2	1	20.70	2	1	1	2	0	0	0	0	0
131	21	2	2	27.46	2	1	2	1	0	0	0	0	0
132	39	2	1	17.94	1	1	1	2	0	0	0	0	0
133	24	2	1	21.86	2	1	2	1	0	0	0	0	0
134	37	2	2	16.31	1	1	2	0	0	0	0	0	0
135	59	2	2	32.05	2	2	2	1	0	0	0	0	0
136	67	1	1	25.26	2	1	2	2	0	1	0	0	0
137	38	2	2	23.12	2	2	1	2	0	0	0	0	0
138	52	2	2	14.63	1	1	1	2	0	0	0	0	0
139	52	2	1	17.47	1	1	1	3	1	0	1	0	0
140	79	1	1	21.69	2	1	2	2	0	0	0	0	0
141	28	2	1	17.20	1	1	2	0	0	0	0	0	0
142	50	2	2	16.41	1	1	1	2	0	1	0	0	0
143	25	2	1	20.76	2	1	2	1	0	0	0	0	0
144	59	2	1	24.73	2	1	1	2	0	0	0	0	0
145	24	2	2	13.29	1	1	2	1	0	0	0	0	0
146	53	2	2	20.18	2	1	2	1	0	0	0	0	0
147	76	1	1	29.64	2	1	2	3	1	0	1	0	0
148	21	2	2	15.78	1	1	1	1	0	0	0	0	0
149	27	2	2	33.88	2	1	2	1	0	0	0	0	0
150	21	2	2	24.36	2	1	2	0	0	0	0	0	0
151	39	2	2	35.12	2	1	1	1	0	0	0	0	0
152	37	2	2	13.58	1	1	2	0	0	0	0	0	0
153	21	2	2	16.09	1	1	1	1	0	0	0	0	0
154	30	2	2	15.44	1	2	2	2	0	0	0	0	0
155	27	2	2	28.11	2	2	2	1	0	0	0	0	0
156	59	2	1	31.93	2	1	1	2	0	1	0	0	0
157	66	1	2	16.61	1	1	1	3	1	0	1	0	0
158	45	2	2	18.87	1	1	1	3	1	0	1	0	1
159	65	1	1	26.52	2	1	2	0	0	1	0	0	0
160	33	2	2	26.09	2	1	2	3	1	0	1	0	0
161	37	2	2	26.94	2	1	2	3	1	0	1	0	0
162	49	2	2	33.12	2	1	2	4	1	0	1	1	1
163	54	2	2	30.11	2	1	2	4	1	0	1	1	1
164	48	2	1	19.19	1	1	1	3	1	0	1	0	1
165	58	2	2	19.71	1	1	2	1	0	0	0	0	0
166	69	1	1	24.78	2	1	2	0	0	0	0	0	0
167	66	1	2	33.00	2	1	1	3	1	0	1	0	1

168	68	1	1	16.99	1	1	1	1	0	0	0	0	0
169	21	2	2	18.47	1	1	2	1	0	0	0	0	0
170	26	2	2	21.37	2	1	2	0	0	1	0	0	0
171	53	2	2	32.37	2	1	2	0	0	0	0	0	0
172	29	2	2	25.96	2	1	2	0	0	0	0	0	0
173	61	1	2	27.54	2	1	1	3	1	0	1	0	1
174	67	1	1	12.14	1	1	1	3	1	0	1	0	1
175	69	1	1	21.82	2	1	2	0	0	0	0	0	0
176	30	2	1	17.53	1	1	2	2	0	1	0	0	0
177	22	2	2	12.98	1	1	2	1	0	1	0	0	0
178	71	1	2	22.23	2	1	1	3	1	0	1	0	0
179	61	1	2	26.46	2	1	2	0	0	0	0	0	0
180	61	1	2	24.44	2	1	1	3	1	0	0	0	0
181	57	2	2	18.07	1	1	2	3	1	1	1	0	0
182	26	2	2	17.43	1	1	2	1	0	0	0	0	0
183	57	2	1	16.54	1	1	1	3	1	0	1	0	0
184	63	1	2	29.62	2	1	2	0	0	0	0	0	0
185	26	2	2	15.82	1	1	2	2	0	0	0	0	0
186	37	2	1	29.25	2	2	1	2	0	0	0	0	0
187	26	2	1	12.21	1	2	2	1	0	0	0	0	0
188	8	2	2	34.02	2	1	2	0	0	0	0	0	0
189	71	1	2	21.32	2	1	2	2	0	0	0	0	0
190	34	2	2	33.02	2	1	2	1	0	0	0	0	0
191	58	2	1	30.40	2	1	1	1	0	0	0	0	0
192	28	2	2	28.65	2	1	1	1	0	0	0	0	0
193	23	2	2	24.47	2	1	2	1	0	0	0	0	0
194	54	2	1	26.72	2	1	2	0	0	0	0	0	0
195	1	2	2	25.14	2	1	2	2	0	0	0	0	0
196	24	2	2	18.20	1	1	2	1	0	0	0	0	0
197	24	2	2	15.92	1	1	2	0	0	0	0	0	0
198	91	1	2	29.53	2	1	2	3	1	1	1	0	0
199	25	2	2	16.7	1	1	2	0	0	0	0	0	0
200	29	2	1	15.21	1	1	2	1	0	0	0	0	0
201	25	2	2	24.02	2	2	2	1	0	0	0	0	0
202	58	2	1	28.34	2	1	1	4	1	0	1	1	0
203	16	2	2	30.50	2	1	2	0	0	0	0	0	0
204	61	1	1	16.88	1	1	2	0	0	1	0	0	0
205	28	2	2	13.30	1	2	2	1	0	0	0	0	0
206	32	2	2	23.21	2	1	2	1	0	0	0	0	0
207	32	2	2	29.44	2	1	2	0	0	0	0	0	0
208	55	2	1	17.49	1	1	2	3	1	0	0	0	0
209	72	1	2	27.16	2	1	1	4	1	0	1	1	1
210	65	1	2	19.04	1	1	1	3	1	0	1	0	1
211	72	1	2	15.05	1	1	1	0	0	0	0	0	0
212	38	2	1	29.58	2	1	2	0	0	0	0	0	0
213	62	1	2	18.55	1	1	2	3	1	0	1	0	0
214	45	2	2	27.50	2	1	2	2	0	0	0	0	0
215	47	2	2	24.33	2	1	1	3	1	0	1	0	0
216	71	1	2	15.63	1	1	1	1	0	0	0	0	0
217	24	2	2	20.50	2	1	2	0	0	0	0	0	0
218	37	2	1	12.47	1	1	2	0	0	0	0	0	0
219	24	2	2	18.65	1	1	2	0	0	0	0	0	0
220	44	2	2	17.40	1	1	2	0	0	0	0	0	0
221	94	1	1	18.30	1	1	2	3	1	0	1	0	0
222	64	1	2	25.01	2	1	1	4	1	0	1	1	0
223	51	2	1	26.21	2	1	2	3	1	0	1	0	0
224	21	2	1	36.79	2	1	2	1	0	0	0	0	0
225	49	2	1	30.32	2	1	1	2	0	1	0	0	0
226	27	2	2	14.19	1	1	1	1	0	0	0	0	0

227	67	1	1	17.52	1	1	1	2	0	0	0	0	0
228	57	2	1	18.32	1	1	2	4	1	0	1	1	1
229	74	1	1	17.47	1	1	2	0	0	1	0	0	0
230	48	2	1	17.57	1	1	1	3	1	0	0	0	0
231	50	2	2	25.79	2	1	2	0	0	0	0	0	0
232	31	2	2	19.43	1	2	2	1	0	0	0	0	0
233	26	2	2	13.93	1	1	2	1	0	0	0	0	0
234	47	2	1	23.71	2	1	1	3	1	0	1	0	1
235	45	2	2	23.66	2	1	1	2	0	1	0	0	0
236	27	2	2	16.49	1	2	1	1	0	0	0	0	0
237	66	1	1	29.95	2	1	1	4	1	0	1	1	1
238	45	2	2	33.36	2	1	2	0	0	0	0	0	0
239	63	1	1	27.7	2	1	2	3	1	0	1	0	0
240	63	1	1	18.44	1	1	2	2	0	0	0	0	0
241	50	2	2	22.63	2	1	1	2	0	0	0	0	0
242	52	2	1	16.38	1	1	1	2	0	0	0	0	0
243	65	1	1	13.97	1	2	1	2	0	0	0	0	0
244	59	2	1	19.29	1	1	2	3	1	0	1	0	0
245	66	1	2	23.73	2	1	1	2	0	1	0	0	0
246	23	2	2	22.67	2	1	2	0	0	0	0	0	0
247	71	1	1	28.46	2	1	1	2	0	0	0	0	0
248	47	2	1	27.27	2	1	2	3	1	1	1	0	0
249	21	2	2	29.90	2	1	1	2	0	0	0	0	0
250	55	2	1	16.70	1	1	2	3	1	0	0	0	0
251	64	1	1	30.1	2	1	2	0	0	1	0	0	0
252	74	1	2	18.07	1	1	2	2	0	0	0	0	0
253	68	1	1	10.93	1	1	2	4	1	0	1	1	0
254	72	1	1	31.32	2	1	1	4	1	0	1	1	0
255	51	2	2	13.62	1	1	2	3	1	0	0	0	1
256	60	2	2	21.24	2	1	1	4	1	0	1	1	0
257	69	1	2	30.05	2	1	1	2	0	0	0	0	0
258	38	2	1	26.25	2	1	2	3	1	0	1	0	0
259	56	2	2	28.74	2	1	1	2	0	0	0	0	0
260	29	2	1	14.40	1	2	2	1	0	0	0	0	0
261	28	2	1	26.63	2	1	2	2	0	0	0	0	0
262	66	1	1	25.05	2	1	1	2	0	0	0	0	0
263	31	2	1	15.11	1	1	2	1	0	0	0	0	0
264	77	1	1	25.40	2	1	1	2	0	0	0	0	0
265	36	2	2	25.40	2	1	2	4	1	1	1	1	0
266	61	1	1	23.16	2	1	2	4	1	0	1	1	1
267	0	2	1	18.69	1	1	2	2	0	0	0	0	0
268	77	1	1	28.01	2	1	1	2	0	0	0	0	0
269	87	1	2	26.60	2	1	1	3	1	0	0	0	0
270	36	2	2	18.89	1	1	2	1	0	0	0	0	0
271	65	1	1	31.97	2	1	1	3	1	0	0	0	0
272	54	2	1	19.20	1	2	1	4	1	0	1	1	0
273	50	2	1	29.58	2	1	1	2	0	0	0	0	0
274	63	1	2	15.14	1	2	1	2	0	0	0	0	0
275	48	2	1	24.01	2	1	2	3	1	1	0	0	0
276	63	1	1	15.80	1	1	1	3	1	1	1	0	0
277	67	1	1	31.06	2	1	1	2	0	1	0	0	0
278	27	2	2	31.24	2	1	2	4	1	0	1	1	0
279	55	2	1	25.56	2	1	1	4	1	0	1	1	1
280	60	2	2	30.51	2	1	1	2	0	1	0	0	0
281	46	2	2	22.32	2	1	1	4	1	0	1	1	1
282	70	1	1	26.22	2	1	1	3	1	0	0	0	1
283	34	2	1	14.73	1	2	1	2	0	0	0	0	0
284	34	2	2	14.73	1	1	2	3	1	0	1	0	0
285	71	1	1	32.25	2	2	1	2	0	0	0	0	0

286	59	2	2	33.59	2	1	1	3	1	0	1	0	0
287	24	2	2	20.50	2	1	2	1	0	1	0	0	0
288	22	2	2	17.33	1	2	2	2	0	0	0	0	0
289	67	1	1	27.87	2	1	1	3	1	1	0	0	0
290	69	1	2	27.50	2	1	1	4	1	0	1	1	0
291	71	1	2	27.87	2	1	1	3	1	0	0	0	0
292	32	2	1	16.38	1	2	2	2	0	0	0	0	0
293	79	1	1	24.16	2	1	1	3	1	0	0	0	1
294	56	2	1	30.55	2	1	1	3	1	0	0	0	0
295	63	1	2	16.56	1	1	1	4	1	0	1	1	0
296	28	2	2	28.91	2	1	2	1	0	0	0	0	0
297	27	2	2	34.16	2	1	2	1	0	0	0	0	0
298	68	1	2	19.56	1	1	1	2	0	1	0	0	0
299	46	2	1	25.76	2	1	1	2	0	0	0	0	1
300	35	2	2	28.71	2	1	2	1	0	0	0	0	0
301	57	2	1	24.09	2	1	1	4	1	0	1	1	1
302	21	2	2	18.77	1	1	1	2	0	0	0	0	0
303	64	1	1	23.40	2	1	1	2	0	1	0	0	0
304	31	2	2	27.07	2	1	1	3	1	0	0	0	0
305	48	2	1	25.22	2	1	1	4	1	0	1	1	1
306	64	1	2	22.08	2	1	1	2	0	1	0	0	0
307	31	2	2	22.65	2	1	2	1	0	0	0	0	0
308	62	1	1	20.43	2	1	1	3	1	0	0	0	0
309	29	2	2	23.45	2	1	1	2	0	0	0	0	0
310	41	2	2	33.20	2	1	1	3	1	0	0	0	0
311	51	2	2	29.50	2	1	1	1	0	1	0	0	0
312	57	2	2	23.66	2	1	1	2	0	1	0	0	0
313	73	1	1	19.56	1	1	2	2	0	0	0	0	0
314	68	1	2	25.03	2	2	1	0	0	0	0	0	0
315	62	1	1	27.10	2	1	1	3	1	1	0	0	0
316	55	2	2	27.33	2	1	1	2	0	0	0	0	0
317	33	2	2	26.98	2	1	1	3	1	0	0	0	0
318	30	2	2	12.76	1	2	2	1	0	0	0	0	0
319	60	2	2	23.21	2	1	1	3	1	0	0	0	1
320	67	1	2	24.96	2	1	1	3	1	0	1	0	0
321	44	2	2	33.07	2	2	2	3	1	0	0	0	0
322	48	2	1	28.30	2	1	1	0	0	1	0	0	0
323	23	2	2	12.95	1	1	2	2	0	0	0	0	0
324	43	2	2	24.92	2	1	1	2	0	0	0	0	0
325	54	2	2	30.11	2	1	1	4	1	0	1	1	1
326	48	2	2	27.02	2	1	1	2	0	0	0	0	0
327	23	2	2	20.61	2	2	2	2	0	0	0	0	0
328	65	1	1	32.14	2	1	2	3	1	1	0	0	0
329	66	1	2	29.44	2	1	1	3	1	0	0	0	0
330	34	2	2	22.75	2	1	1	2	0	0	0	0	0
331	28	2	2	18.21	1	1	2	1	0	0	0	0	0
332	55	2	2	29.56	2	1	2	0	0	0	0	0	0
333	64	1	1	28.14	2	2	2	0	0	0	0	0	0
334	33	2	2	19.40	1	1	2	0	0	0	0	0	0
335	32	2	2	9.80	1	1	2	1	0	0	0	0	0
336	60	2	2	17.09	1	1	1	2	0	1	0	0	0
337	61	1	1	26.92	2	2	1	1	0	0	0	0	0
338	24	2	2	21.46	2	1	2	1	0	0	0	0	0
339	84	1	1	19.51	1	1	1	3	1	0	0	0	1
340	63	1	1	17.38	1	2	2	0	0	0	0	0	0
341	67	1	1	28.03	2	1	1	3	1	1	0	0	0
342	70	1	1	27.28	2	1	1	1	0	0	0	0	0
343	49	2	2	18.00	1	1	2	2	0	0	0	0	0
344	51	2	1	24.39	2	1	1	2	0	1	0	0	0

345	61	1	2	27.45	2	1	1	3	1	0	0	0	0
346	35	2	1	23.94	2	1	1	4	1	0	1	1	0
347	55	2	2	29.99	2	1	1	2	0	0	0	0	0
348	38	2	2	16.21	1	1	2	0	0	0	0	0	0
349	64	1	2	18.92	1	1	1	2	0	0	0	0	0
350	20	2	2	32.13	2	1	2	1	0	0	0	0	0
351	32	2	2	16.28	1	2	2	2	0	1	0	0	0
352	48	2	2	29.75	2	1	1	3	1	0	0	0	0
353	44	2	2	32.47	2	1	1	3	1	0	0	0	1
354	45	2	1	24.10	2	1	1	3	1	0	0	0	1
355	57	2	1	24.12	2	1	1	4	1	0	1	1	1
356	35	2	1	29.99	2	1	1	2	0	0	0	0	0
357	35	2	2	13.12	1	1	2	0	0	0	0	0	0
358	27	2	2	23.38	2	1	2	2	0	0	0	0	0
359	48	2	1	20.05	2	1	1	3	1	0	0	0	1
360	50	2	2	15.46	1	2	2	2	0	1	0	0	0
361	63	1	2	18.79	1	1	1	2	0	0	0	0	0
362	70	1	2	15.25	1	1	1	2	0	0	0	0	0
363	80	1	1	31.52	2	1	1	4	1	0	1	1	1
364	32	2	2	16.31	1	1	1	0	0	0	0	0	0
365	36	2	2	34.36	2	2	2	2	0	0	0	0	0
366	61	1	2	23.64	2	1	1	3	1	0	0	0	0
367	60	2	1	30.49	2	1	2	2	0	0	0	0	0
368	29	2	1	11.23	1	2	1	2	0	1	0	0	0
369	64	1	1	19.16	1	2	1	0	0	0	0	0	0
370	35	2	2	18.80	1	1	1	1	0	0	0	0	0
371	38	2	1	13.50	1	2	1	2	0	0	0	0	0
372	74	1	2	19.05	1	1	2	2	0	1	0	0	0
373	34	2	1	18.53	1	2	2	2	0	0	0	0	0
